# Increased biofilm formation in dual-strain compared to single-strain communities of *Cutibacterium acnes*

**DOI:** 10.1038/s41598-024-65348-y

**Published:** 2024-06-24

**Authors:** Cecilie Scavenius Brønnum Bjerg, Anja Poehlein, Mechthild Bömeke, Axel Himmelbach, Andreas Schramm, Holger Brüggemann

**Affiliations:** 1 Department of Biomedicine, Faculty of Health, Aarhus University, Aarhus, Denmark; 2 Department of Biology, Section for Microbiology, Aarhus University, Aarhus, Denmark; 3 Department of Genomic and Applied Microbiology, Institute of Microbiology and Genetics, University of Göttingen, Göttingen, Germany; 4 Leibniz Institute of Plant Genetics and Crop Plant Research (IPK), Gatersleben, Germany

**Keywords:** *Cutibacterium acnes*, Biofilm; polymicrobial infection, Orthopedic implant-associated infection, Transcriptome, Microbiology, Bacteria, Bacteriology, Biofilms, Microbial genetics, Pathogens

## Abstract

*Cutibacterium acnes* is a known opportunistic pathogen in orthopedic implant-associated infections (OIAIs). The species of *C. acnes* comprises distinct phylotypes. Previous studies suggested that *C. acnes* can cause single- as well as multi-typic infections, i.e. infections caused by multiple strains of different phylotypes. However, it is not known if different *C. acnes* phylotypes are organized in a complex biofilm community, which could constitute a multicellular strategy to increase biofilm strength and persistency. Here, the interactions of two *C. acnes* strains belonging to phylotypes IB and II were determined in co-culture experiments. No adverse interactions between the strains were observed in liquid culture or on agar plates; instead, biofilm formation in both microtiter plates and on titanium discs was significantly increased when combining both strains. Fluorescence in situ hybridization showed that both strains co-occurred throughout the biofilm. Transcriptome analyses revealed strain-specific alterations of gene expression in biofilm-embedded cells compared to planktonic growth, in particular affecting genes involved in carbon and amino acid metabolism. Overall, our results provide first insights into the nature of dual-type biofilms of *C. acnes*, suggesting that strains belonging to different phylotypes can form biofilms together with additive effects. The findings might influence the perception of *C. acnes* OIAIs in terms of diagnosis and treatment.

## Introduction

*Cutibacterium acnes* is a Gram-positive bacterium commonly found on human skin, in particular in sebaceous gland-rich areas like the face and upper back^1^. Although generally considered beneficial, *C. acnes* can become an opportunistic pathogen and is associated with diseases such as acne vulgaris and orthopedic implant-associated infections (OIAIs)^2–5^. The biofilm-forming ability of *C. acnes* on various prosthetic materials contributes to its role as an opportunistic pathogen and biofilm formation likely increases the tolerance of *C. acnes* to antimicrobial agents^6–11^.

The *C. acnes* species is divided into three subspecies: subsp. *acnes* (I), subsp. *defendens* (II), subsp. *elongatum* (III); six different phylotypes (IA_1_, IA_2_, IB, IC, II, and III) can be distinguished based on core genome differences^4,5^. Over the last decade, new tools have made it easier to identify *C. acnes* phylotypes, e.g. single- and multi-locus sequence typing (SLST and MLST) schemes^12,13^. However, the significance of different phylotypes in disease formation remains to be elucidated. Commonly identified *C. acnes* phylotypes associated with OIAIs are type IB and type II, but also type IA_1_ strains have been detected^14–20^. Recent studies using culture-dependent and -independent approaches showed that multi-typic *C. acnes* OIAIs exist, i.e. infections caused by strains belonging to more than one phylotype^21,22^; they may even account for the majority of *C. acnes* OIAIs^22^.

To date, there is a lack of knowledge regarding multi-typic biofilms and the interactions of different *C. acnes* phylotypes in such biofilms. Understanding the potential interactions of *C. acnes* is crucial, as a multi-typic infection could pose a greater risk to the patient, and mutualistic interactions between *C. acnes* phylotypes could explain why some infections are difficult to treat. In line with this, a study reported that mono- and multi-typic *C. acnes* OIAIs appear to be two different clinical entities with different clinical histories and immune responses^17^. Therefore, a detailed description of biofilm dynamics and bacterial interactions between *C. acnes* phylotypes is needed.

In this study, we investigated the interactions and biofilm formation of single- and dual-type communities of *C. acnes* strains isolated from OIAIs. We could show that biofilm formation for the investigated strains is increased in dual-strain compared to single-strain communities, and the two investigated *C. acnes* strains belonging to different phylotypes have distinct changes in gene expression when grown in biofilms.

## Results

### Selection of strains

Two clinical *C. acnes* isolates that had been obtained in a previous study from the same elbow OIAI were selected^22^: EASDk81A, belonging to subsp. *acnes*/phylotype IB; and EASDk81B, belonging to subsp. *defendens*/phylotype II (Table 1). We used Nanopore sequencing to close the genomes, resulting in circular chromosomes of 2.560 Mb and 2.497 Mb for strains EASDk81A and EASDk81B, respectively. To further investigate the relatedness of the two strains with other *C. acnes* strains a core genome comparison was done with *C. acnes* genomes available in public databases (Supplementary Fig. S1). It could be confirmed that EASDk81A belongs to phylotype IB/SLST class H and EASDk81B belongs to phylotype II/SLST class K.

**Table 1:** Features of the two *C. acnes* strains used in this study.

	*C. acnes* EASDk81A	*C. acnes* EASDk81B
Subspecies	*acnes*	*defendens*
Phylotype	IB	II
SLST type	H1	K8
Genome size (Mbp)	2.560652	2.497202
GC content (%)	60	60
Genes (CDS)*	2443 (2362)	2394 (2315)
Coding sequences (% of genome)	96.68%	96.70%
Strain-specific CDS**	264 (11.2%)	218 (9.4%)

*Predicted by Prokka.

**BlastP with the cutoffs: 50% sequence coverage and 50% protein sequence identity.

**Figure 1:**
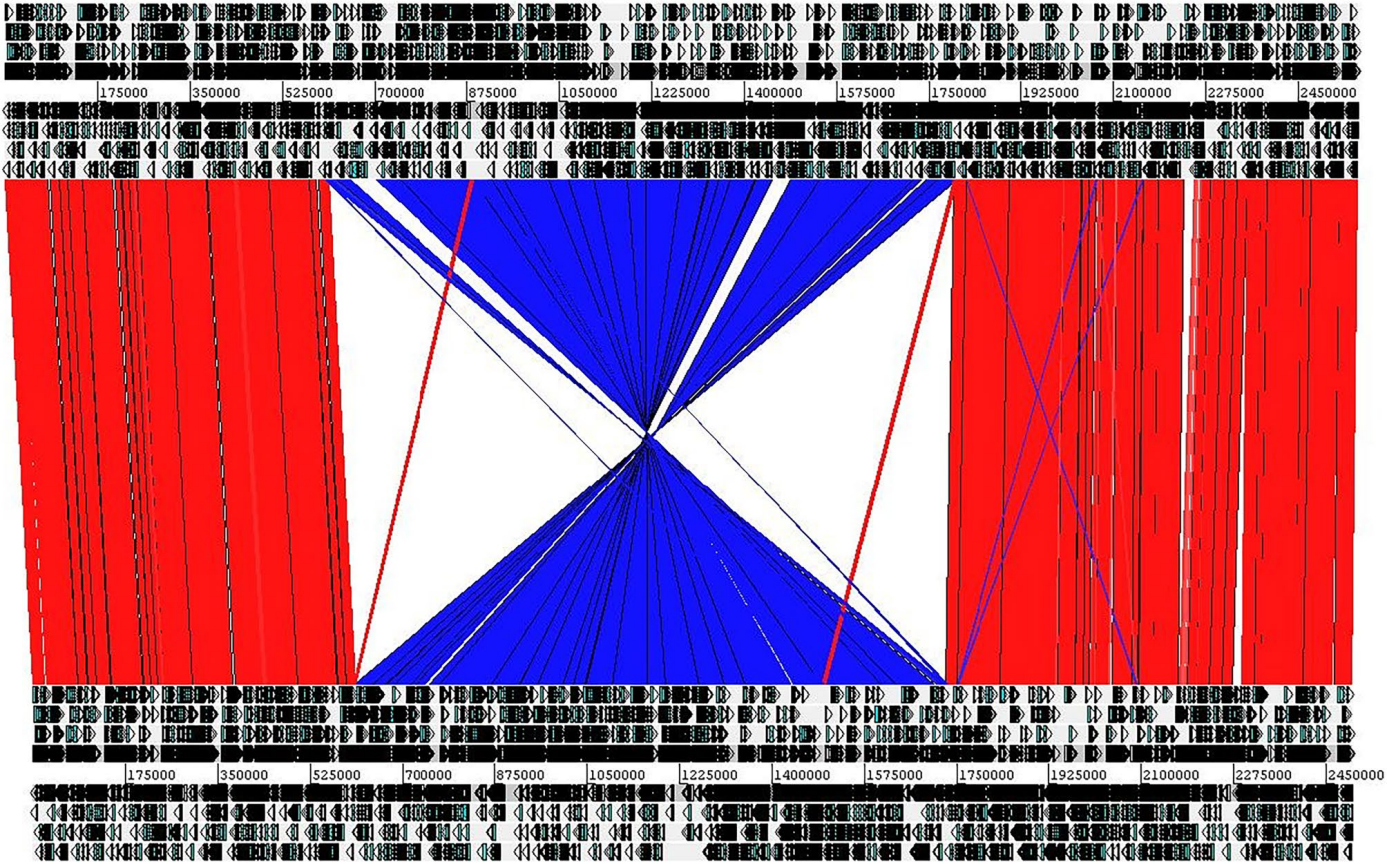
Comparison of the closed chromosomes of the two *C. acnes* strains EASDk81A and EASDk81B. The chromosomes of strains EASDk81A (top) and EASDk81B (bottom) were compared with the Artemis comparison tool (ACT). A large 1.1 Mb inversion between the two strains was identified (in blue). Strain-specific regions are scattered around the chromosomes.

When comparing the in-silico predicted proteomes of the two strains, 264 and 218 strain-specific coding sequences (CDS) could be identified in EASDk81A and EASDk81B, respectively, encoded in strain-specific genomic loci (Table 1). Genome comparison and alignment revealed a large DNA inversion of 1.1 Mb between the two chromosomes (Fig. 1). The average nucleotide identity (ANI) of the core genome (alignment of 92% of the full genome) was 97.42%. In total, 53,487 single nucleotide variants (SNVs) were identified in the core genome of the two strains; thus, in average, ca. 23 SNVs were present per 1000 bp.

### No detectable negative interferences of the two *C. acnes* strains on solid and in liquid media

In order to investigate possible interferences, the two strains were tested against each other in an antagonistic plate assay, where each of the strains was used as indicator strain or stab culture, respectively. For both strains, no growth inhibition could be detected as judged from the absence of any clearing zone in the lawn strain around the stab culture (Supplementary Fig. S2).

**Figure 2:**
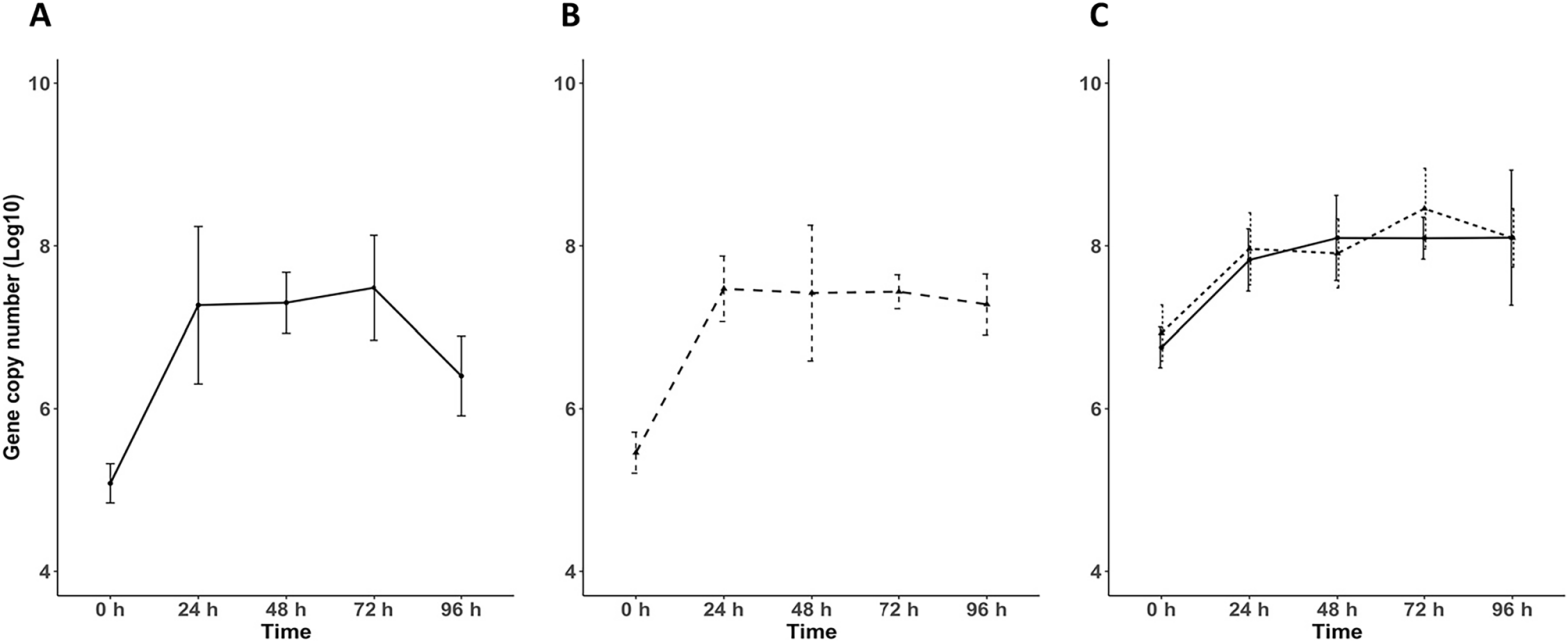
Gene copy numbers in mono- and co-cultures of the two *C. acnes* strains EASDk81A and EASDk81B. Samples were taken after 0 h, 24 h, 48 h, 72 h, and 96 h and analyzed by qPCR. A) monoculture of strain EASDk81A (type IB); B) monoculture of strain EASDk81B (type II); C) co-culture of strains EASDk81A (solid line) and EASDk81B (dashed line). A limitation of this experiment was the difficulty to reach equal starting cell numbers based in OD measurements. Error bars represent means ± SD of three replicates.

In a second experiment, batch culture experiments with mono- and co-cultures in BHI broth were performed. To monitor the individual growth of each strain in the co-culture, copy numbers of strain-specific genes were quantified by qPCR (Fig. 2). Gene copy numbers of both strains were detectable at similar numbers in the co-culture, with a lack of gene copy number decrease of the two strains when grown in co-cultures until 96 h, indicating that there were no growth interferences between the two strains in broth culture.

### Increased biofilm formation in dual- compared to single-type cultures of *C. acnes*

**Figure 3:**
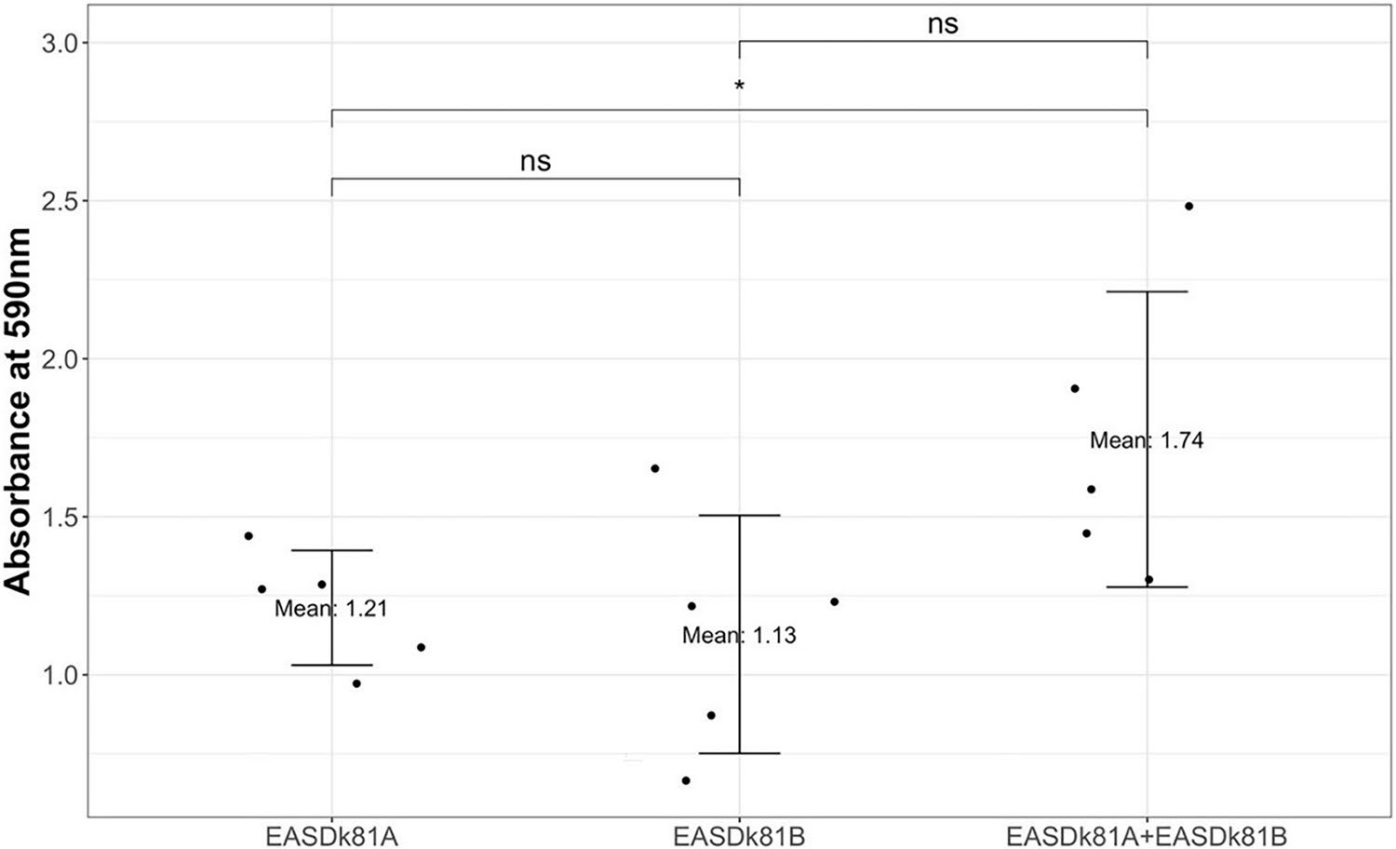
Quantification of biofilm formation in single- and dual-type cultures of *C. acnes*. Biofilm formation was determined in a microliter plate assay using crystal violet staining after 48 h of incubation. An additive effect in dual-type compared to single-type cultures was observed. The experiment was replicated five times for each single- and dual-type culture. Statistical analysis was performed using the R Wilcoxon test (*p < 0.05).

We wanted to compare the biofilm formation of *C. acnes* strains EASDk81A and EASDk81B in mono- and co-culture, hereafter called single- and dual-type cultures, respectively. Biofilm formation was quantified and visualized by staining with crystal violet after 48 h of incubation in a microtiter plate assay. Results showed an increase in biofilm formation of the dual-type culture compared to the single-type cultures, indicative of an additive effect of co-cultivation (Fig. 3). This increase was statistically significant (*p* < 0.05) for the comparison of the dual-type culture and the single-type culture of strain EASDk81A only.

**Figure 4:**
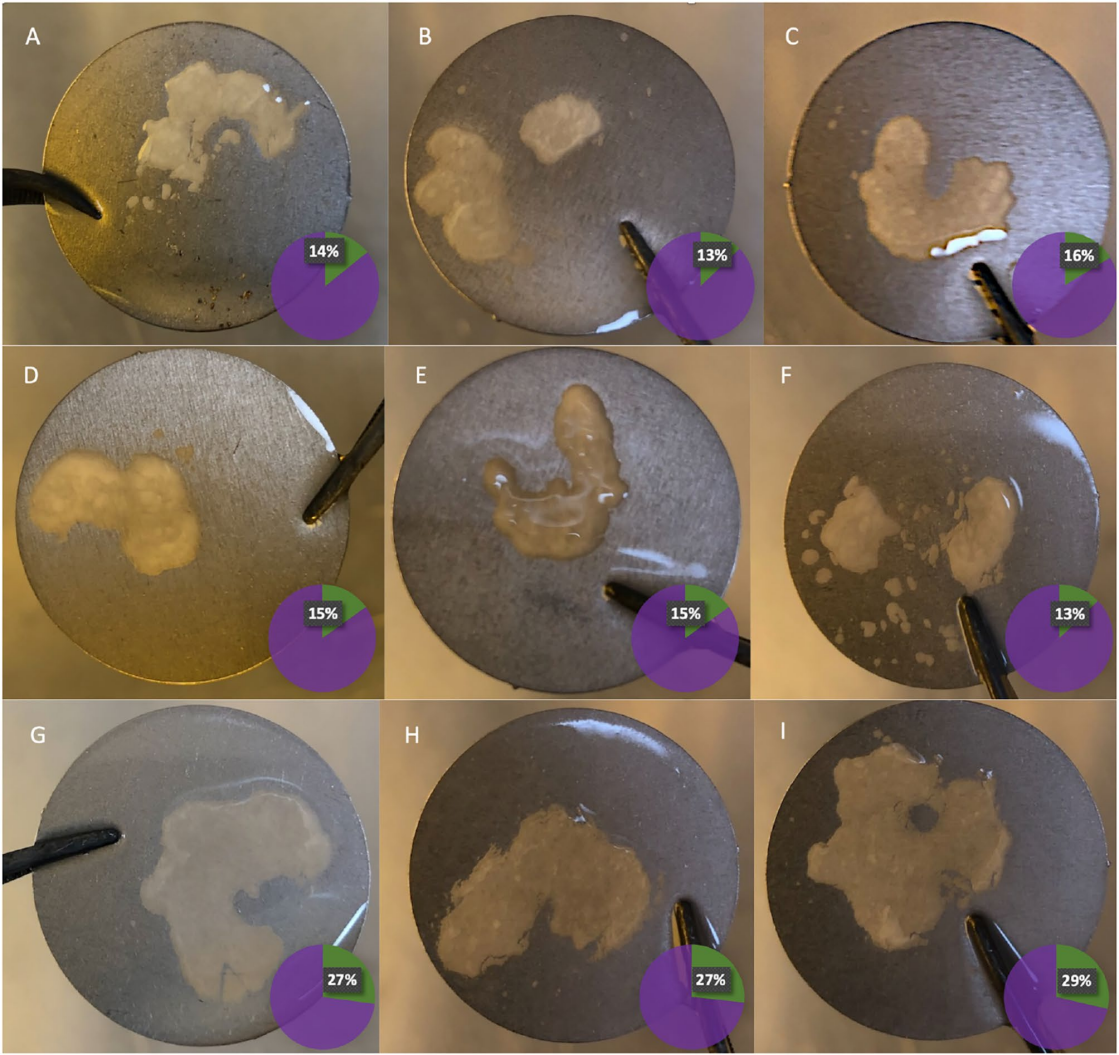
Biofilm formation of *C. acnes* in single- and dual-type cultures on titanium discs. Biofilm formation on titanium discs was determined after 96 h of incubation. A-C, monoculture of strain EASDk81A; D-F, monoculture of strain EASDk81B; G-I, co-culture of strains EASDk81A and EASDk81B. All discs were washed to remove non-adherent cells. Area analysis was done using ImageJ; results are shown in pie charts with the percentage of biofilm coverage of the total area of the disc given.

Biofilm formation was assessed in a more clinically relevant model system, using titanium discs. The biofilm-covered area on the discs was quantified in single- and dual-type cultures after four days of incubation (Fig. 4); the average coverage of the disc surface by single-type biofilms of strains EASDk81A and EASDk81B was 14.3 ± 1.5% and 14.3 ± 1.1%, respectively (mean ± SD; n = 3). For dual-type biofilms, the average coverage was 27.7 ± 1.1%. Thus, the biofilm-covered region on titanium discs was almost twice as large in dual-type compared to single-type cultures.

### Visualization of the *C. acnes* two-strain community biofilm

**Figure 5:**
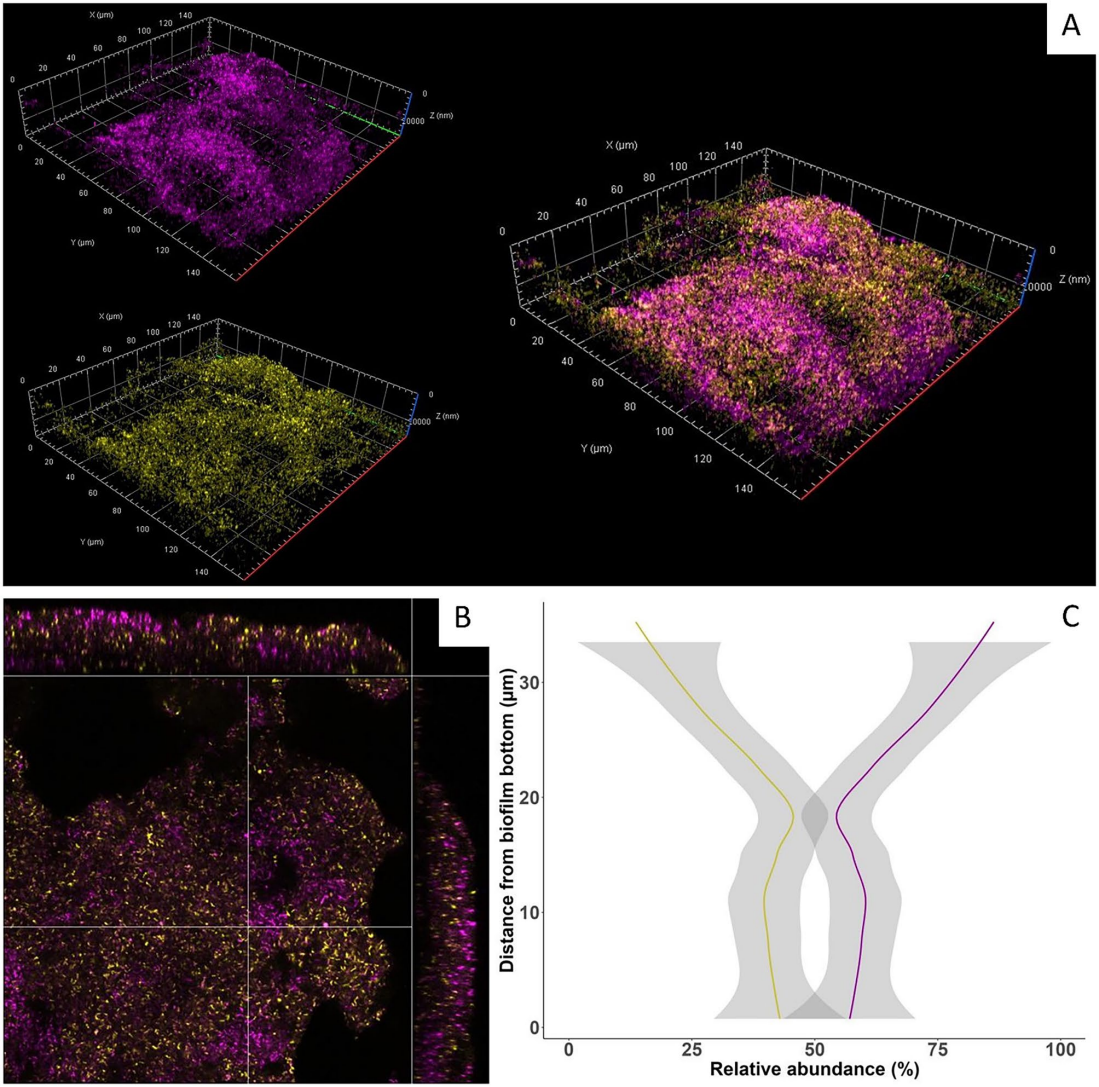
Confocal images and spatial analysis of the dual-type biofilm of *C. acnes*. Fluorescence in-situ hybridization was applied using strain-specific probes. Yellow, strain EASDk81A; purple, strain EASDk81B. (**A**) 3D visualization of z-stack images taken through the biofilm from top to bottom of the disc. Left, separate images for each probe; right, overlay of both channels. (**B**) Perpendicular xy-, xz-, and yz-planes through randomly selected points in the biofilm. XY image is 160 × 160 µm and shown at 15 µm depth in the biofilm. (**C**). Spatial analysis of the dual-type biofilm. The biofilms were created in triplicates and z-stack images were analyzed, resulting in relative abundances of EASDk81A (yellow) and EASDk81B (purple). Statistical analysis utilized R package geom_smooth, and error envelopes are displayed for both strains.

Confocal laser scanning microscopy images of dual-type biofilms after FISH revealed the spatial distribution of the two strains EASDk81A and EASDk81B (Fig. 5). The strains were in close proximity, with EASDk81B forming more compact focal structures than EASDk81A. There was no strong overgrowth of one strain over the other in the dual-type biofilm, albeit strain EASDk81B was overall more abundant, in particular in the upper layers of the biofilm, while the strains were more equally distributed at the bottom of the biofilm (Fig. 5C).

### Comparative transcriptome analysis of *C. acnes* EASDk81A and EASDk81B as planktonic and biofilm-embedded cells

Gene expression of strains EASDk81A and EASDk81B was investigated when grown as single-type planktonic cultures and as single-type biofilms. Biofilm samples were taken from titanium discs. We could not perform gene expression analysis of the two strains grown in a dual-type biofilm, since unambiguous RNA-seq read mapping was not possible, due to the high sequence similarity of the genomes of the two strains (97.42% average nucleotide identity of the core genome).

**Figure 6:**
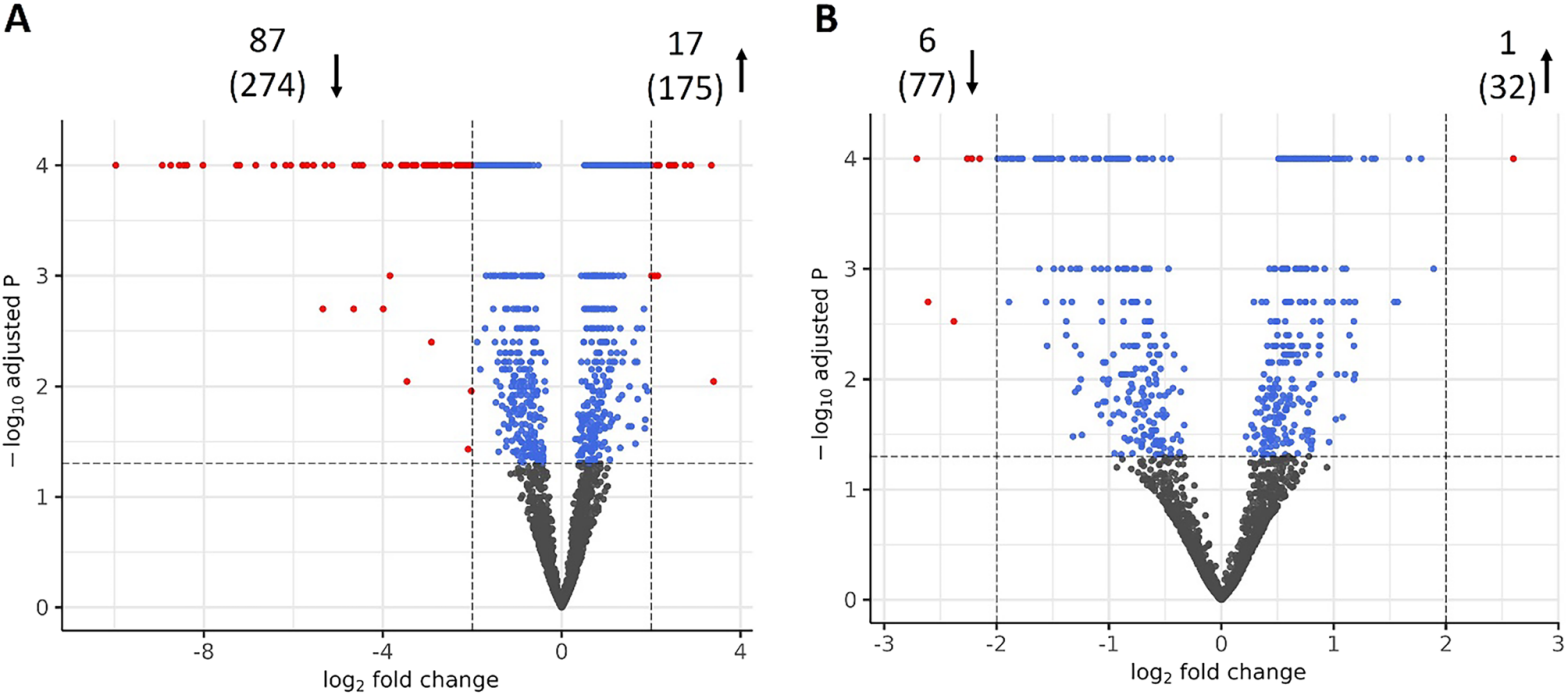
Transcriptome analysis of *C. acnes* growing as planktonic cultures and in single-type biofilms. (A) Differentially expressed genes (DEGs) in *C. acnes* EASDk81A grown as single-type planktonic culture versus single-type biofilm. (**B**) DEGs in *C. acnes* EASDk81B grown as single-type planktonic culture versus single-type biofilm. DEGs with a log_2_-fold change > 2 or < −2 are highlighted in red and numbers of DEGs are given. In brackets given the DEGs with a log_2_-fold change > 1 or < −1 (i.e., at least twofold up- or downregulated genes, respectively). The data is based on three biological replicates.

Differentially expressed genes (DEGs) between planktonic and biofilm-grown cells for EASDk81A and EASDk81B were determined (Fig. 6; Supplementary Tables S1 and S2). The strain EASDk81A underwent substantial changes in transcription in the transition from planktonic to biofilm growth with 17 and 87 at least fourfold up- and downregulated genes, respectively. The transcriptional changes in strain EASDk81A affected several genes involved in energy metabolism, e.g. downregulation of the F-type ATPase and the upregulation of the fumarate reductase and the cytochrome bd complex. An apparent shift in the metabolism was also seen by the 5.3-fold upregulation of a catabolite repressor protein (CRP)-like cAMP-activated global transcriptional regulator. This regulator might be involved in carbon catabolite repression (CCR), in analogy to its function in a number of bacterial species^23^. Metabolic adaptation went along with the deregulation of genes involved in amino acid biosynthesis (21 genes downregulated), co-factor biosynthesis (9 and 7 genes up- and downregulated, respectively) and substrate transport (ABC transporters and phosphotransferase systems (PTS); 9 and 12 genes up- and downregulated, respectively). Thus, strain EASDk81A adapted substantially to the different conditions in the biofilm compared to planktonic growth. In contrast, in strain EASDk81B only 1 and 6 genes were strongly (fourfold or more) up- and downregulated, respectively, in biofilm-embedded cells compared to planktonic growth, indicative of limited adaptive changes.

## Discussion

*Cutibacterium acnes* is a common human skin bacterium with proposed beneficial roles for human health. However, it can also cause OIAIs, in particular shoulder OIAIs^24,25^. Some studies have found evidence for polymicrobial OIAIs involving *C. acnes*, but also monomicrobial OIAIs with multiple *C. acnes* strains belonging to different phylotypes (i.e. multi-typic infections) have been described^17,21,22^. Since clinical laboratories usually do not test for multi-typic *C. acnes* infections, the proportion of such infections among OIAIs is currently unknown. Our recent data suggests that the majority of *C. acnes* OIAIs are actually multi-typic infections^22^.

Here, we investigated the interactions between two OIAI isolates of *C. acnes* belonging to different phylotypes. *C. acnes* strains EASDk81A (type IB) and EASDk81B (type II) were selected for this study as they were co-isolated from the same implant infection^22^. They belong to two different *C. acnes* subspecies (subsp. *acnes* and subsp. *defendens*) and have different morphologies and hemolytic activities, i.e. EASDk81A showed hemolysis on blood-agar plates, while EASDk81B did not^22^.

First, solid and liquid media experiments did not reveal any negative or positive interference between the two strains. Strain-specific qPCR analyses in liquid culture over time showed that both strains grew in the co-culture in similar quantities. The data also indicated that co-culturing might have resulted in a longer survival in the stationary phase of the strains, compared to growth in monocultures.

Then, two different biofilm assays were applied. As previously noted, microtiter plate assays do not mimic the in vivo scenario accurately^11,26^. Thus, in addition to microtiter plate assays we used titanium discs in our biofilm experiments that better simulate the conditions of a prosthetic implant surface. Previous studies have indicated that *C. acnes* can form biofilms on this type of material^27^, and our results showed that both strains could attach to the surface and form biofilms on the discs. Interestingly, when growing together, the two-strain community increases biofilm production in the microtiter plate assay as well as in the titanium disc assay compared to monocultures. This indicates that the two *C. acnes* strains co-operate in biofilm formation. Visualization with the FISH technique showed the spatial organization of the biofilm. The two strains were closely interconnected in the biofilm, but the data further suggested layering and cluster formation within the biofilm. Both strains could be detected at similar levels in the lower part in close proximity (0–20 µm) to the titanium disc surface the primary attachment site. However, strain EASDk81B was more abundant in the upper layers of the biofilm (> 20 µm from the surface). This might indicate that strain EASDk81B can attach to the primary biofilm or can attach to EASDk81A cells more easily.

In order to investigate underlying mechanisms of strain interaction and cooperation, transcriptome analyses were carried out and revealed gene expression differences between planktonic and sessile *C. acnes* cells. Some interesting findings are discussed here.

In both *C. acnes* strains, DEGs were mostly downregulated in the transition from planktonic growth to biofilm embedment (5–6 times more downregulated than upregulated DEGs (Supplementary Tables S1 and S2), indicative of a reduced metabolic activity in the biofilm. This could suggest a transition to dormancy or quiescence, which is a hallmark of persister cells. Persister cells, which are usually more tolerant to antibiotics, can be found in biofilms formed by other bacterial species, such as *Staphylococcus aureus*^28,29^.

The number of DEGs in strain EASDk81A was 15 times higher than in strain EASDk81B during the transition from planktonic growth to biofilm embedment. This could indicate that the type II strain EASDk81B is more adjusted to biofilm conditions per se, or the strain has limited regulatory power to adapt to the sessile environment compared to strain EASDk81A. There was very limited overlap between the DEGs of EASDK81A and EASDk81B, indicating that the two strains responded differently when grown in a biofilm.

Several DEGs in strain EASDk81A in the transition from planktonic growth to biofilm embedment were involved in energy metabolism, including the upregulation of the fumarate reductase, which is an important system to gain energy during propionate production by the Wood-Werkman cycle^30^. A main shift in its general metabolism during biofilm embedment of strain EASDk81A is also indicated by the 5.3-fold upregulation of a gene encoding a homolog of a CRP-like cAMP-activated global transcriptional regulator. CRPs are important global regulators and involved in CCR in a number of bacterial species^23^. In addition, CRPs are also involved in regulating biofilm formation^31,32^. Possibly linked to CRP-dependent CCR is the differential regulation of 21 genes encoding ABC transporters and PTS systems. This could indicate that strain EASDk81A is able to adapt to changing carbon sources. Another metabolic gene strongly upregulated in EASDk81A in the biofilm state encodes a ribonucleotide reductase (RNR) of class III (anaerobic), essential for the synthesis of the four deoxyribonucleotides necessary for DNA replication and repair. Different classes of RNRs exist and it was previously shown that they are differentially expressed in biofilms of *E. coli*^33^.

In contrast, the strongest upregulated gene in strain EASDk81B grown in biofilm compared to planktonic growth encodes a putative cysteine synthase (CysK). In *E. coli* and other bacteria, CysK generates l-cysteine from *O*-acetyl l-serine and hydrogen sulfide. CysK was found to be the key enzyme in the cysteine biosynthetic pathway and involved in promoting biofilm formation in *Vibrio fischeri*^34^. However, the exact role of CysK in biofilm formation is unknown. It was suggested that CysK might function in biofilm formation on two levels, one of which is to provide cysteine^34^.

In both strains the cytochrome bd complex of the respiratory chain was upregulated in biofilm-embedded *C. acnes*. The cytochrome bd complex is one of the two terminal oxidases in the respiratory chain of many bacterial species, catalyzing the reduction of O_2_ to H_2_O. The enzyme is specially used under low oxygen tension^35^. Surprisingly, this oxidase is also present in some anaerobes and “nanaerobes” such as *C. acnes,* i.e. organisms that do not require oxygen for growth, but can benefit from the presence of nanomolar concentrations of oxygen. This indicates that the cytochrome bd complex has an additional function and it was indeed recently discovered that the complex contributes to bacterial protection against toxic small molecules such as hydrogen peroxide, nitric oxide, and hydrogen sulfide^36^. Since growth conditions in our experiments were anoxic, it is possible that the upregulation of cytochrome bd complex in biofilm-embedded *C. acnes* has a protective function against the damaging effects of toxic small molecules. Another possible defense mechanism is the enzyme nitric-oxide reductase (NOR); the gene was 5.7-fold upregulated in strain EASDk81A in the biofilm state compared to planktonic growth. NOR can protect against nitric oxide toxicity^37^.

Biofilm-specific differential regulation of genes encoding virulence factors of *C. acnes* was investigated, despite the fact that knowledge regarding virulence factors of *C. acnes* is still limited. Genes coding for a putative lysophospholipase (n35_22230) and a hyaluronate lyase (n35_03920) were found to be 3.6- and 2.6-fold upregulated, respectively, in biofilm conditions versus planktonic growth in strain EASDk81A (Supplementary Table S1). Both proteins have been identified as secreted factors in *C. acnes* strain KPA171202^38^; the hyaluronate lyase has been studied and found to be important for hyaluronic acid degradation^39,40^. It can be speculated that strain EAS81Dk81A has increased its tissue-invading potential in biofilm conditions due to the enhanced production of these tissue component-degrading enzymes. In contrast, in strain EASDk81B the expression of known or putative virulence factor genes was not altered during the transition to the biofilm state. For example, the strongly expressed CAMP factor 1 in strain EASDk81B (n36_13760) was not differently regulated in planktonic versus biofilm growth. A previous study has found different results^41^: it reported the slight upregulation of CAMP factors 1 and 4 (twofold and 1.6-fold, respectively) of *C. acnes* strain KPA171202 (a type IB strain) in biofilm compared to planktonic growth. However, in the respective study, besides using a different *C. acnes* strain, different growth conditions were applied and biofilms were grown on plastic surface (T-25 cell culture flasks) with an incubation of six days.

## Conclusion

The study aimed to investigate biofilm formation and bacterial interactions in dual-strain biofilms produced by *C. acnes* strains isolated from OIAI. The results showed signs of bacterial interactions between clinical isolates of *C. acnes,* here the type IB strain EASDk81A and the type II strain EASDk81B. This cooperation resulted in a significant increase in biofilm formation. To what extent these results can be generalized for other type IB and type II strains and combinations thereof (and even for strains belonging to other phylogenetic clades of *C. acnes*) needs to be experimentally tested in the future.

Overall, the findings suggest a synergistic relationship between the *C. acnes* strains as a multicellular strategy to enhance biofilm formation and persistence. The study provided new insights into behavioral adaptations of *C. acnes* phylotypes in a community setting and emphasizes the complexity of multi-typic infections. These findings could have an impact on current diagnostic procedures and treatment regimens against *C. acnes* OIAIs.

## Materials and methods

### Selection of strains and bacterial growth conditions

Two clinical isolates, *C. acnes* EASDk81A (type IB, H1) and *C. acnes* EASDk81B (type II, K8), were used in this study, obtained in a previous study^22^ (Table 1). They both originated from the same OIAI case (elbow OIAI) and were obtained by cultivation of the sonication fluid of the respective implant. Reinforced Clostridial Agar (RCA) was used as the agar-based growth medium for the *C. acnes* strains. Brain Heart Infusion (BHI) broth was used as the growth medium in all liquid culture experiments, and BHI medium supplemented with 10% human plasma (Sigma Aldrich, St. Louis, USA) was used in the titanium disc biofilm assay. The incubation in all experiments was carried out anaerobically at 37 °C.

### Genome sequencing

For genomic DNA extraction, the MasterPure™ Gram-Positive DNA Purification Kit (Lucigen, Middleton, USA) was used as per manufacturer’s instructions. Illumina sequencing of EASDk81A and EASDk81B has been done previously^22^ and the genome sequences are stored in GenBank with the accession numbers JAJCVV000000000 and JAJCVU000000000, respectively.

Nanopore sequencing was applied to close the draft genomes. 1.5 μg unsheared DNA was used for the library preparation using the ligation sequencing kit 1D (SQK-LSK109) and the native barcode expansion kit (EXP-NBD103). Sequencing was performed for 72 h on a MinION Mk1B device with a SpotON R9.4.1 flow cell, using MinKNOW v22.05.5 and Guppy v6.2.1 in high accuracy mode for base calling (Oxford Nanopore, Oxford, UK). Unicycler v0.5.0^42^ was used to perform the hybrid assembly (Illumina and Nanopore sequence reads), resulting in one circular replicon per strain. The closed genomes are stored in Genbank with the accession numbers CP129003 (EASDk81A) and CP129004 (EASDk81B).

### Core genome comparison

*C. acnes* genomes with a high sequence quality (contig N50 > 500 kb) were taken from GenBank (n = 262, including 43 closed *C. acnes* genomes; status May 2024). Core genome comparison and single nucleotide variants (SNVs) were determined with the Parsnp program from the Harvest software package (v1.7.4)^43^. Core-genome-based phylogeny was reconstructed based on core-genome SNVs using FastTree 2^44^.

### Antagonistic agar plate assay

Liquid cultures in BHI medium were prepared of both *C. acnes* strains grown for 48 h with shaking at 37°C under anaerobic conditions. Both cultures were adjusted to OD_600_ 0.1 by diluting with BHI medium after which 1 mL of the bacterial suspension of strain EASDk81A was distributed on a RCA plate (the indicator strain). The plate was completely air-dried. 10 µl of the other liquid culture (strain EASDk81B) was used as stab culture and pipetted on top of the indicator strain plate and incubated for 24–48 h in anaerobic conditions. Antagonistic assays were conducted using both strains as both indicator strains and stab cultures.

### Bacterial co-culture

The main cultures were incubated for 48 h with shaking of 120 rpm at 37°C under anaerobic conditions. The cultures were then adjusted to OD_600_ 0.1 by diluting with BHI medium. Cultures were mixed to a total volume of 50 ml. Single and mixed cultures were incubated at 37°C anaerobically with shaking. Samples were taken in an anaerobic chamber at 24 h, 48 h, 72 h and 96 h. The sample was centrifuged for 5 min at 1500 g at 4°C, and the pellet was used for DNA extraction.

### Microtiter plate test

Biofilm microtiter plate assays were made as follows. Main cultures in BHI broth were prepared (48 h, anaerobic incubation). Cultures were adjusted to 0.1 at OD_600_ before being mixed to a final volume of 2 ml. Sterile polystyrene flat-bottomed 96-well plates were inoculated with 120 µl of bacterial suspension and incubated anaerobically with shaking for 24 h before the supernatant was removed, and 120 µl BHI medium was added. Plates were then incubated for additional 48 h. For determining biofilm formation, wells were washed with phosphate-buffered saline (PBS), and the plates were air-dried. Adherent cells were fixed with 120 µl ethanol (99%), followed by crystal violet staining where 100 µl of crystal violet solution (0.5%) was added to each well and incubated at room temperature for 20 min. After 20 min, excess crystal violet was removed by carefully washing the plate. Subsequently, the remaining dye bound to the adherent cells was released by adding 100 µl of ethanol (96%). The absorbance was measured at 590 nm using a plate reader.

### Titanium-disc biofilm assay

For the titanium disc biofilm assay, pure titanium discs Grade 2 ASTM-B265 (2.54 cm diameter, 0.0508 cm thickness) were purchased (Sigma Aldrich, St. Louis, USA). The discs were soaked in 70% ethanol for 20 min and air-dried until completely dry, followed by autoclaving at 121 °C for 30 min. BHI medium supplemented with 10% human plasma (Sigma Aldrich, St. Louis, USA) were used for all experiments. The plasma was received freeze-dried and was resuspended in 5 ml of sterile milliQ water; it was subsequently sterile filtrated through a 0.2 µm filter.

The main cultures were made in BHI medium for 48 h of incubation. Cultures were adjusted to OD_600_ 0.1 by diluting with BHI medium. The cultures were used as single-type cultures and mixed cultures (EASDk81A and EASDk81B) with a total volume of 10 ml. In a 6-well culture plate, a titanium disc was placed with sterile forceps in each well before adding 1 ml of bacterial suspension followed by 2 ml of BHI medium supplemented with plasma (10%). Six wells were used as technical replicates, and three biological replicates were made. The plates were incubated in a shaking incubator with 50 rpm of shaking under anaerobic conditions at 37°C for 96 h. This incubation time was empirically chosen and resulted in the strongest biofilms on titanium discs compared to other incubation times. After the incubation, the discs were removed and washed in NaCl (0.9%) three times. The discs were either used for FISH or the biofilm was harvested for RNA sequencing.

### qPCR

#### Construction of standards for qPCR

Single copy strain-specific genes were selected, i.e. *lanB* (EASDk81A) and *cas3* (EASDk81B). Fragments of the genes were amplified by PCR, using specific primer sets (Supplementary Table S3) and with a reaction mixture consisting of 9.5 µl sterile DNA free water, 2 µl primer mix, 1 µl of 1:100 diluted genomic DNA of the respective *C. acnes* strain and 12.5 µl AccuStartII PCR Supermix (Taq polymerase). Amplification was carried out at 35 cycles at the following PCR conditions: an initial step of 94 °C for 40 s for early denaturation, then denaturation at 94 °C for 35 s followed by primer annealing at 55°C for 40 s elongations at 72°C for 40 s and a final elongation for 7 min at 72 °C. The primers were tested against the two strains and determined to be strain-specific, i.e. the *lanB* primers only resulted in a product in strain EASDk81A and the *cas3* primers only gave a product in strain EASDk81B. All products were visualized on a 1% agarose gel. The PCR products were purified with the GenEluteTM PCR Clean-Up Kit (Sigma Aldrich, St. Louis, USA) according to the manufactures protocol. The DNA concentration of the PCR products was quantified with the Qubit dsDNA high-sensitivity assay (Qubit 4.0 Fluorometer, Invitrogen, Carlsbad, USA). The purified PCR product was ligated into the plasmid pGEM®-T vector system (Promega, Madison, USA) using the cloning kit containing rapid ligation buffer, pGEM®-T vector, and T4-DNA ligase. Ligation was performed according to the manufacturer’s protocol. The ligated plasmid DNA was transformed into competent *E. coli* JM 109 cells using the heat shock method. Luria Broth (LB) plates containing ampicillin (50 μg/ml) and IPTG (0.5 mM)/X-Gal (80 μg/ml) for blue-white screening were prepared. The ligation mixture was plated out and plates were incubated overnight at 37°C. White colonies were checked with a colony-PCR (vector-specific primers M13-F (5’-GTAAAACGACGGCCAG-3’) and M13R (5’-GTCATAGCTGTTTCCTG-3’)), to verify the correct cloning of the respective gene fragments. In addition, Sanger sequencing (Macrogen Europe, Amsterdam, The Netherlands) was done to ensure the correct amplification without mutations.

The DNA concentration of the PCR products was quantified with the Qubit dsDNA high-sensitivity assay (Qubit 4.0 Fluorometer, Invitrogen, Carlsbad, USA). The DNA concentration was converted to gene copies/µl prior to making the standards. This was done by taking the DNA concentration measurements of the purified plasmid and incorporating them in the equation below.

$$ \frac{{\left( {6.023 \cdot 10^{23} } \right){*}\left( {{\text{DNA }}\;{\text{conc}}.{ }\left( {\frac{{\text{g}}}{{{\mu L}}}} \right)} \right)}}{{{\text{the}}\;{\text{ size}}\;{\text{ of}}\;{\text{ the}}\;{\text{ insert}}\; \left( {{\text{bp}}} \right) \cdot 660\left( {\frac{{\text{g}}}{{{\text{mol}}}}} \right)}} = Gene\; copy\; number\; \left( {\mu L} \right) $$

The gene copy number was calculated, and serial dilutions were made so the dilutions would cover the largest range of reactions (1 $\cdot$ 10^8^–1 $\cdot$ 10^1^ copies/µL).

#### qPCR in co-cultures

In co-culture experiments samples were taken at 0 h, 24 h, 48 h, 72 h, and 96 h. DNA extraction from pellets obtained from the co-culture experiments was conducted using Lucigen MasterPure ™ Gram Positive DNA Purification Kit, following the manufacturer’s protocol (Lucigen, Middleton, USA). The qPCR reactions utilized the reagent mix protocol to quantitatively amplify the two strain-specific gene fragments (*lanB* and *cas3*). The reaction mix protocol for qPCR reactions contained 10 μl of 2 × RealQ Plus 2 × Master mix Green Low ROX (AMPLIQON), 1 µL of each primer (10 pmol), 2 μl of bovine serum albumin (10 μg/mL), 2 µl of DNA template and DNA free water to a reaction volume of 20 μl. qPCR amplification of the two *C. acnes* strain-specific gene fragments was carried out at 40 cycles with the following PCR conditions: an initial step at 95°C for 15 min, then 35 cycles of 95°C for 30 s, primer annealing at 55°C for 30 s, the elongation step at 72°C for 20 s, and data acquisition at 80°C for 5 s. qPCR was done on a AriaMx Real-time PCR system (Agilent, Santa Clara, USA)**.** R-squared values ranged between 0.995 and 1, and amplification efficiency ranged between 95–98%. The qPCR reactions were run in triplicates using strain-specific standard dilutions, DNA templates, and a negative amplification control. The results were analyzed by assessing the standard curves of the threshold cycle (Ct) vs. the log of gene copy number.

### Fluorescence in situ hybridization

#### Probe design

We designed 18 bp oligonucleotide probes (Supplementary Table S3) targeting a region of the *C. acnes* 23S rRNA that contains a single-nucleotide polymorphism (SNP) differentiating strain EASDk81A and EASDk81B. Probe performance and hybridization conditions were analyzed in silico using mathFISH^45^. The probes were labelled with the fluorescent dyes Atto488 and Atto542, respectively. Hybridization conditions were tested experimentally on pure cultures of EASDk81A and EASDk81B (fixed with 50% ethanol in PBS), and 25% formamide in the hybridization buffer were required to reliably differentiate the two strains.

#### FISH on biofilm samples

After biofilm formation on the titanium discs, the discs were placed in 50% ethanol in PBS for fixation. The discs were air-dried before dehydrating in a series of ethanol solutions (50%, 80%, and 100%) for 3 min in each solution. After dehydration, the discs were air-dried before enzyme treatment. Enzyme treatment was done with both lysozyme (10 mg/mL) for 60 min followed by achromopeptidase (60 U/mL) for 30 min. After each treatment, the discs were washed with dH2O and 96% ethanol. Hybridization was done at 46°C for 90 min as previously described using 25% formamide in the standard FISH buffer^46^. The discs were then mounted in 10 µl Citiflour (AF3)/Vectashield (4:1) and imaged on an LSM 800 Airyscan Laser Scanning Confocal microscope with super-resolution (ZEISS, Jena, Germany).

#### Image analysis

The analysis of the confocal images was done using the Daime analysis program (v. 2.2.3)^47^. Analysis of biofilm covered area on the titanium discs was done using ImageJ (v. 1.53) by following the protocol of measuring and counting objects.

### RNA extraction, transcriptome sequencing and analysis

For RNA seq analysis, cells from planktonic cultures and cells from single- and dual-type biofilms were harvested. For biofilm harvesting, the titanium discs were washed on ice and placed in a petri dish with 1.5 ml of cold BHI medium on ice. Next, the discs were sonicated at 40% (80 W) in an ice bath for 10 min and vortexed shortly before the pellet was spun down at 1500 g for 5 min at 4 °C. Harvested bacteria were resuspended in 800 μl RLT buffer (RNeasy Mini Kit, Qiagen, Hilden, Germany) with β-mercaptoethanol (10 μl/ml) and cell lysis was performed using a laboratory ball mill. Subsequently, 400 μl buffer RLT (RNeasy Mini Kit Qiagen) with β-mercaptoethanol (10 μl/ml) and 1,200 μl 96% [v/v] ethanol were added. For RNA isolation, the RNeasy Mini Kit (Qiagen, Hilden, Germany) was used, following the instructions of the manufacturer, but instead of buffer RW1, the buffer RWT (Qiagen, Hilden, Germany) was used in order to also isolate RNAs smaller than 200 nt. To determine the RNA integrity number (RIN) the isolated RNA was run on an Agilent Bioanalyzer 2100 using an Agilent RNA 6000 Nano Kit, as recommended by the manufacturer (Agilent Technologies, Waldbronn, Germany). The remaining genomic DNA was removed by digestion with TURBO DNase (Invitrogen, Thermo Fisher Scientific, Paisley, UK). The Illumina Ribo-Zero plus rRNA Depletion Kit (Illumina, San Diego, USA) was used to reduce the amount of rRNA-derived sequences.

For sequencing, strand-specific cDNA libraries were constructed with a NEBNext Ultra II directional RNA library preparation kit for Illumina and the NEBNext Multiplex Oligos for Illumina (New England BioLabs, Frankfurt, Germany). To assess the quality and size of the libraries, samples were run on an Agilent Bioanalyzer 2100 using an Agilent High Sensitivity DNA Kit, as recommended by the manufacturer (Agilent Technologies, Waldbronn, Germany). The concentration of the libraries was determined using the Qubit® dsDNA HS Assay Kit, as recommended by the manufacturer (Life Technologies GmbH, Darmstadt, Germany). Sequencing was performed on a NovaSeq 6000 instrument (Illumina, San Diego, USA) using NovaSeq 6000 SP Reagent Kit v1.5 (100 cycles) and the NovaSeq XP 2-Lane Kit v1.5 for sequencing in the paired-end mode and running 2 × 50 cycles. For quality filtering and removing of remaining adaptor sequences, Trimmomatic-0.39^48^ and a cutoff phred-33 score of 15 was used. Mapping against the reference genome was performed with Salmon (v 1.5.2)^49^. As mapping backbone, a file that contained all annotated transcripts excluding rRNA genes and the whole genome sequence of the reference as a decoy was prepared with a k-mer size of 11. Decoy-aware mapping was done in selective-alignment mode with “–mimicBT2,” “–disableChainingHeuristic,” and “–recoverOrphans” flags as well as sequence and position bias correction. For –fldMean and –fldSD, values of 325 and 25 were used, respectively. The quant. sf files produced by Salmon were subsequently loaded into R (v 4.0.3) using the tximport package (v 1.18.0)^50^. DeSeq2 (v 1.30.0)^51^ was used for normalization of the reads; fold-change shrinkages were also calculated with DeSeq2 and the apeglm package (v 1.12.0)^52^.

### Supplementary Information

Supplementary Information 1.

Supplementary Information 2.

Supplementary Information 3.

Supplementary Information 4.

Supplementary Information 5.

## Data Availability

The transcriptome data has been stored in the SRA database with the accession number PRJNA987963. Genome sequences have been stored in Genbank with the accession numbers CP129003 (EASDk81A) and CP129004 (EASDk81B).
