# Supplementary figures and images for: Increased biofilm formation in dual-strain compared to single-strain communities of Cutibacterium acnes
Source: Sci Rep. 2024 Jun 24;14:14547. doi: 10.1038/s41598-024-65348-y (PMC11196685; doi:10.1038/s41598-024-65348-y)

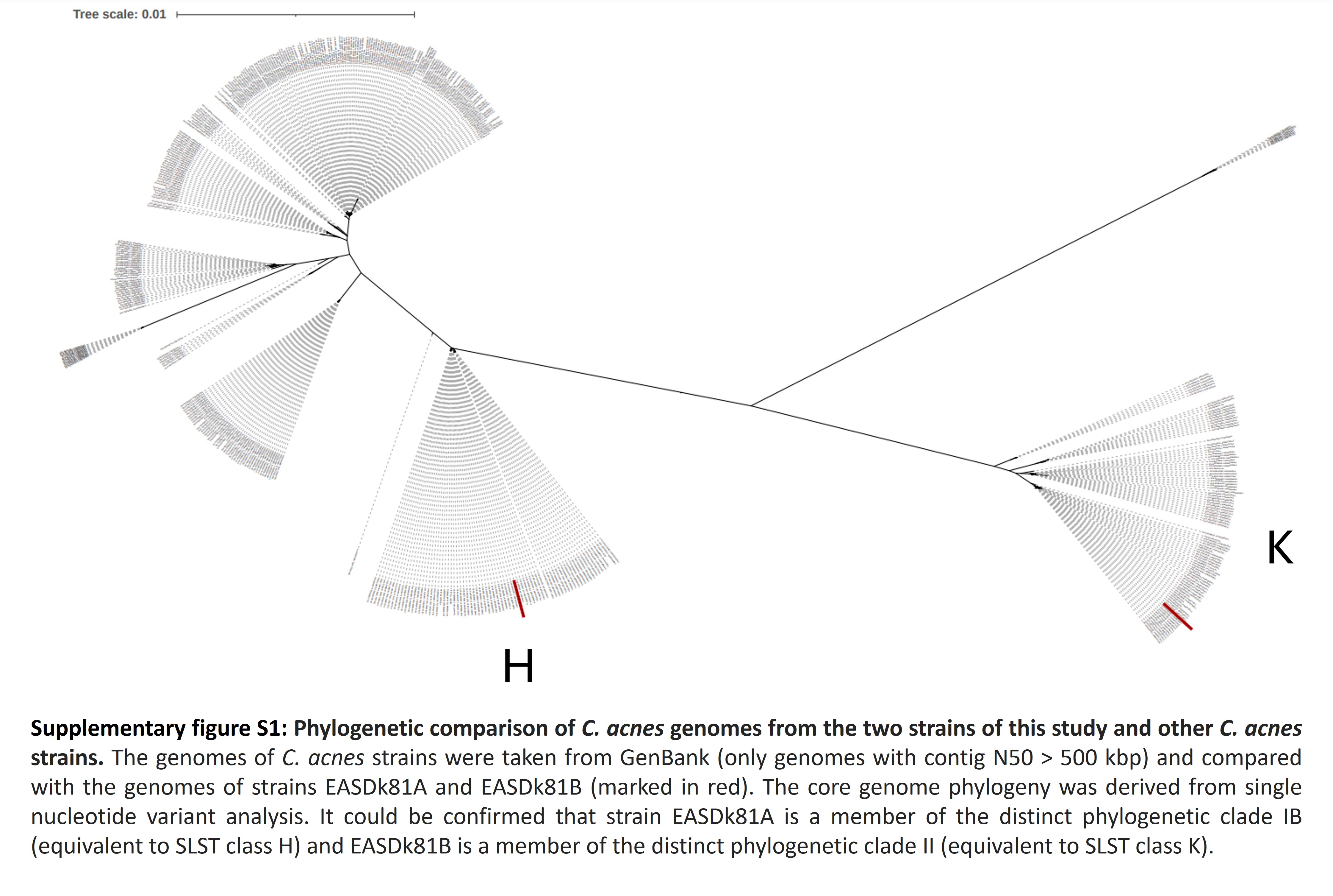

Supplement: Supplementary file 1 — Supplementary Information 1. [file 41598_2024_65348_MOESM1_ESM.jpg]

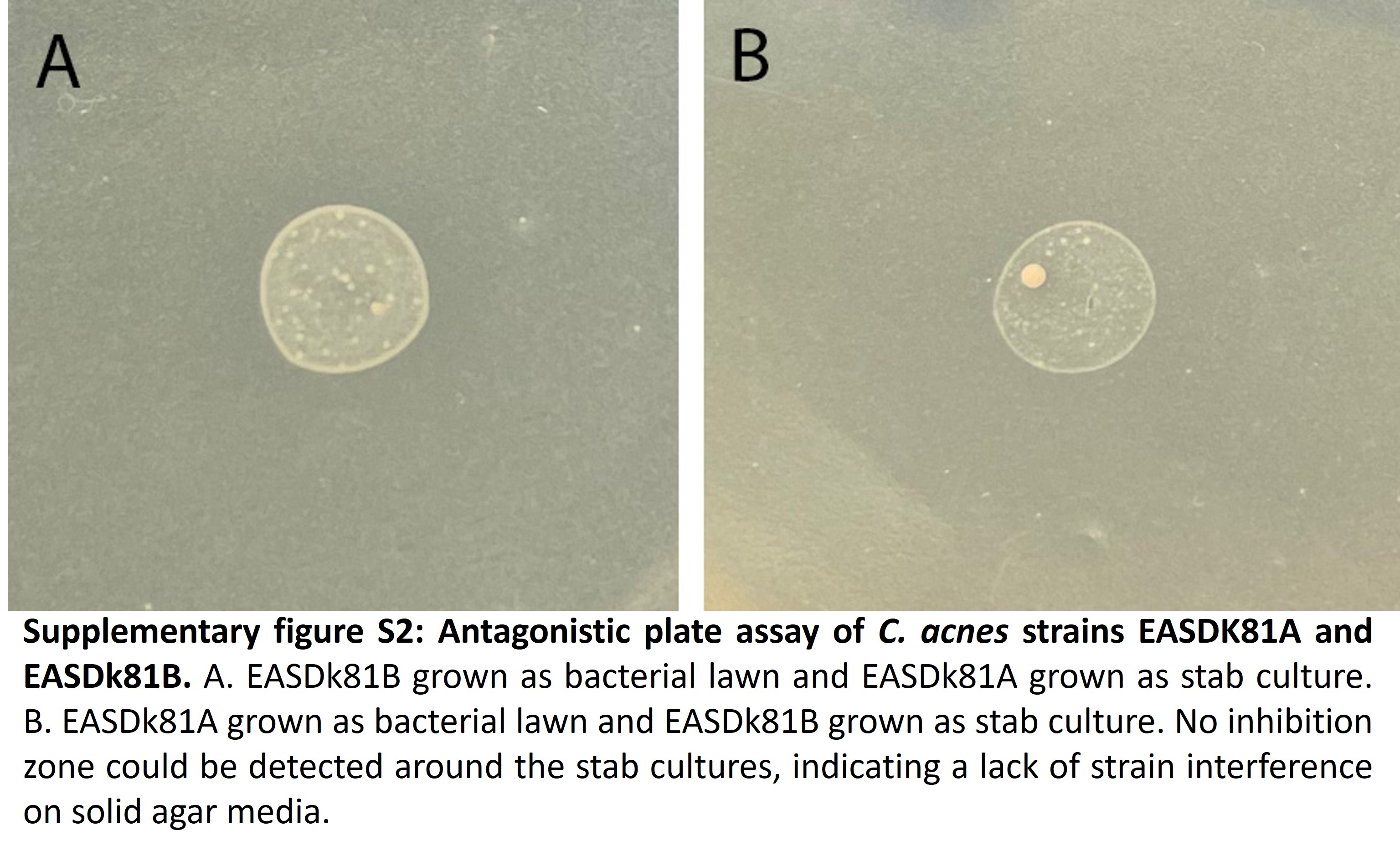

Supplement: Supplementary file 2 — Supplementary Information 2. [file 41598_2024_65348_MOESM2_ESM.jpg]
