# Supplementary material for: Increased biofilm formation in dual-strain compared to single-strain communities of Cutibacterium acnes
Source: Sci Rep. 2024 Jun 24;14:14547. doi: 10.1038/s41598-024-65348-y (PMC11196685; doi:10.1038/s41598-024-65348-y)
Supplement: Supplementary file 5 — Supplementary Information 5. [file 41598_2024_65348_MOESM5_ESM.docx]

**Supplementary table S3. Primers and probes used in this study**

| Primer/probe | Gene | *C. acnes* strain | purpose | name |
| --- | --- | --- | --- | --- |
| 5’-GTTTTCGCGCCTTAGCATTC-3’  5’-GAGATTCAAGGTGGGCTTCG-3’ | *lanB* | EASDk81A | qPCR | LanB_for  LanB_rev |
| 5’-ATCCCTGGGACATCGATCAG-3’  5’-GAATTTGGCGATCGAGAAGG-3’ | *cas3* | EASDk81B | qPCR | Cas3_for  Cas3_rev |
| 5’CCCACAACCATGGGCTCC3’ | 23S rRNA | EASDk81A | FISH | Pac81A-1712_atto488 |
| 5’CCCACAACCGTGGGCTCC3’ | 23S rRNA | EASDk81B | FISH | Pac81B-1712_atto542 |
